# Anatomy of the atria: A road map to the left atrial appendage

**DOI:** 10.1007/s00399-017-0535-x

**Published:** 2017-11-03

**Authors:** Umberto Barbero, Siew Yen Ho

**Affiliations:** 1 Cardiology Unit, Città della Salute e della Scienza Hospital, University of Turin, Turin, Italy; 2 Cardiac Morphology Unit, Royal Brompton and Harefield NHS Foundation Trust and Imperial College London, SW3 6NP London, UK

**Keywords:** Heart atria, Atrial appendage, Atrial fibrillation, Puncture, Occlusion, Herzvorhöfe, Herzohr, Vorhofflimmern, Punktion, Verschluss

## Abstract

The left atrial appendage (LAA) has received increasing attention in recent years because of thrombi formation in patients with atrial fibrillation, which increases the risk of stroke. In patients who have contraindications for long-term oral anticoagulation therapy, percutaneous procedures are used to occlude the LAA and there are now several devices available for implantation, both endocardially and epicardially. Despite the high-resolution imaging techniques on hand today, limitations remain in providing information about wall thickness and neighboring structures; therefore, in-depth knowledge of the normal atrial anatomy is mandatory when considering such interventions. Here, the anatomy of the right and left atria is reviewed with relevance to interventional procedures required for LAA occlusion. The components of the atria, particularly the LAA as well as the atrial septum, are described with emphasis on their spatial relationships to neighboring cardiac and extracardiac structures. Sound knowledge of the atrial anatomy including endocardial and epicardial aspects is necessary. This will help interventionists take full advantage of imaging techniques when assessing the suitability of the LAA anatomy for closure, selecting the optimal device types and sizes, and guiding the LAA closure procedure, thereby reducing potential complications and increasing procedural success.

## Zusammenfassung

Das linke Herzohr („left atrial appendage“, LAA) erhielt in den letzten Jahren verstärkte Aufmerksamkeit wegen Thrombenbildung bei Patienten mit Vorhofflimmern, was das Risiko eines Schlaganfalls erhöht. Bei Patienten mit Kontraindikationen für eine Langzeitantikoagulationstherapie werden perkutane Interventionen zum Verschluss des LAA genutzt. Mittlerweile sind verschiedene Systeme zur Implantation verfügbar, sowohl endokardial als auch epikardial. Trotz heute vorhandener hochauflösender Bildgebungsverfahren bestehen weiterhin Einschränkungen hinsichtlich Informationen über die Wanddicke und benachbarte Strukturen, daher ist bei Erwägung derartiger Interventionen ein detailliertes Wissen über die normale Vorhofanatomie Voraussetzung. In der vorliegenden Arbeit wird die Anatomie des rechten und linken Vorhofs dargestellt sowie ihre Bedeutung für interventionelle Verfahren, die zum LAA-Verschluss erforderlich sind, erörtert. Die Bestandteile der Vorhöfe, insbesondere des LAA und des Vorhofseptums, werden mit Betonung auf ihre räumlichen Beziehungen zu benachbarten kardialen und extrakardialen Strukturen beschrieben. Eine gründliche Kenntnis der Vorhofanatomie einschließlich ihrer endokardialen und epikardialen Aspekte ist notwendig. Dies trägt dazu bei, dass die Operateure das Potenzial bildgebender Verfahren voll ausschöpfen können, wenn sie die Eignung des LAA für eine Verschlussintervention beurteilen, optimale Gerätetypen und -größen auswählen und die LAA-Verschlussintervention leiten; somit werden potenzielle Komplikationen vermindert und der Operationserfolg gesteigert.

## Introduction

The left atrial appendage (LAA) is a highly complex structure that has received increasing attention in recent years, primarily because over 90% of thrombi in patients with atrial fibrillation (AF) occur here [1], which increases the risk of stroke threefold [2]. To manage this complication, percutaneous LAA occlusion has evolved [3] and European guidelines for management of AF now recommend it in patients at high risk for stroke who have contraindications for long-term oral anticoagulation (Class IIb Indication, Level of Evidence B) [4].

Previously, the LAA was ligated, stapled, or amputated during elective open-heart surgery. Surgical techniques continue to evolve with the type of sutures used and the delivery of clips through sternotomy or thoracotomy. In recent years, however, percutaneous procedures are increasingly used and there are now several devices available for implantation within the LAA to occlude its orifice. In addition, there is an epicardial LAA exclusion system that enables percutaneous suture ligation of the LAA via combined pericardial and transseptal access [5, 6]. There are more closure devices undergoing trials or in development [7]. Each of these implants, whether delivered endocardially or epicardially, have different characteristics and exclusion criteria for fitting and occluding the LAA. High-resolution imaging techniques can now be used to study the anatomy of the LAA [8], but there are still limitations in providing information about wall thickness and neighboring structures. Therefore, knowledge of the left atrium and the LAA anatomy is mandatory for interventional cardiologists performing LAA closure to facilitate understanding of the images, choosing the device, and preventing complications. Equally important is the anatomical understanding of the right atrium and the atrial septum, which constitute the endocardial access route to the LAA. In addition, the operator should pay close attention to the spatial relationship of the atrial chambers to structures neighboring the epicardial surface.

## The right atrium

**Fig. 1:**
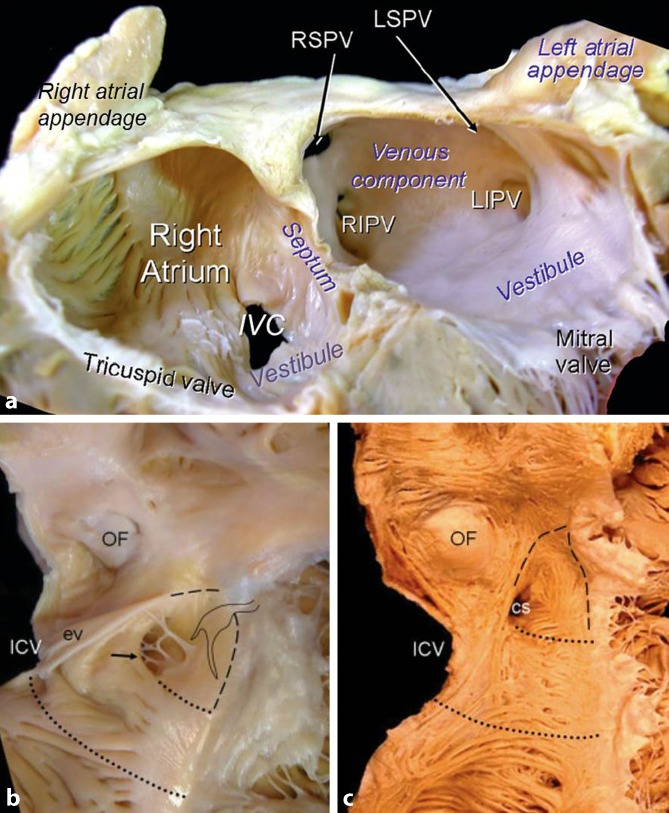
**a** Dissection of the atria following removal of their anterior walls viewed from a left anterior perspective to show their components. **b** Right lateral view of the opened right atrium shows the oval fossa (*OF*) and its surrounding rim as well as the landmarks for the triangle of Koch. The orifice of the coronary sinus (*arrow*) is guarded by a fenestrated thebesian valve. The short, broken line continuing from the free edge of the eustachian valve (*ev*) marks the tendon of Todaro, while the hinge line of the tricuspid valve marks the anterior border (*long broken line*). At the apex lies the membranous septum (*pale area*). The irregular shape marks the anticipated site of the compact atrioventricular node with its inferior extensions. The *short, dotted line* marks the “septal” isthmus, and the *long, dotted line* marks the cavo-tricuspid isthmus. **c** This specimen is displayed in similar orientation and it has been dissected to show the subendocardial myocardial architecture. The landmarks for the triangle of Koch and isthmuses are superimposed. *cs* coronary sinus, *ICV* and *IVC* inferior caval vein, *L* and *RIPV* left and right inferior pulmonary vein, *L* and *RSPV* right and left superior pulmonary vein. (Fig. 1a reproduced from [14], with permission by AHA; Fig. 1b and 1c reproduced from [26], with permission by Wiley)

When delivering catheters into the heart, the first chamber encountered is the right atrium. The atrium is best considered in terms of three components: the venous part, the appendage, and the vestibule (Fig. 1a). The remaining wall is the septum shared with the left atrium, which per se is crucial for the interventional cardiologist to have a firm understanding of its anatomy.

The eustachian valve, guarding the entrance of the inferior caval vein, is a variably developed flap. Usually it is a triangular flap of fibrous or fibromuscular tissue that inserts medially to the eustachian ridge (or sinus septum), which is the border between the oval fossa and the coronary sinus. Occasionally it is a filigreed mesh, or a perforated flap, which can be so extensive as to stretch across the superior caval vein orifice and is described as Chiari’s network [9]. The free border of the eustachian valve continues into the musculature of the sinus septum, as a fibrous strand called the tendon of Todaro [10]. It is of particular importance, being one of the borders of Koch’s triangle that delineates the anatomical landmarks for the location of the atrioventricular node (Fig. 1b, c). The hinge line of the septal leaflet of the tricuspid valve marks the anterior border of the triangle, while the inferior border is the orifice of the coronary sinus, with the atrioventricular node located superiorly within the triangle’s apex [11]. A small, usually fenestrated, crescentic flap, the thebesian valve, guards the orifice of the coronary sinus. The latter might be dilated, suggesting the presence of a persistent left superior vena cava draining into the coronary sinus [12] or, rarely, anomalous pulmonary venous connection. The terminal crest (crista terminalis) is a muscle band that marks the lateral and posterior border between the pectinated wall of the appendage and the smooth wall of venous component. Pectinate muscles arise from the crest to line the endocardial surface of the appendage. The atrial walls in between the branching pectinate muscles are exceptionally thin with only a few strands of myocytes sandwiched between epi- and endocardial surfaces. The anterolateral course of the terminal crest at the entrance of the superior vena cava is the landmark for the location of the sinus node [13].

## The atrial septum

The atrial septum separates the atrial chambers (Figs. 1a **and** 2). Although the atrial chambers are designated right and left, the atrial septum does not run in the anterior–posterior orthogonal plane. Instead, it is obliquely orientated such that the left atrium is situated somewhat posterior to the right atrium. Structurally, this component is more complex than it appears at first glance [14]. The right atrial view of the septal aspect can be misleading because it gives the impression of an extensive septum.

**Fig. 2:**
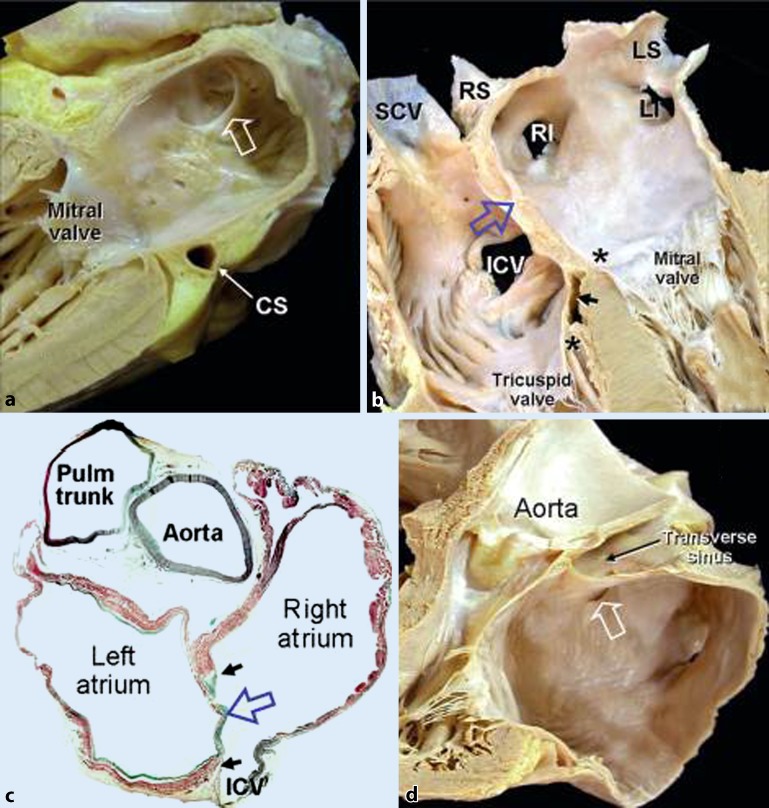
**a** Longitudinal cut through the left atrium and left ventricle showing the endocardial surface of the septal wall of the atrial component indistinguishable from the anterior and posterior walls other than for the crescent-like margin (*open arrow*) that marks the site of the patent foramen ovale if it is persistent. Note the location of the coronary sinus (*CS*) relative to the inferior wall. **b** Longitudinal cut through the four cardiac chambers showing the atrial septum in profile. The floor of the oval fossa (*open arrow*) is the true septum. *Asterisks* mark the levels of attachments of the tricuspid and mitral valves at the septum. The inferior pyramidal space (*small arrow*) is covered by the vestibule of the right atrium. **c** Histological section taken through the short axis of the heart showing the thin flap valve (*open arrow*) and the muscular rim of the fossa (*small arrows*). Note the uneven thickness of the left atrial wall. **d** View of the septal component showing a patent foramen ovale (*open arrow*). Its opening is behind the anterior wall of the left atrium and the transverse pericardial sinus. *ICV* inferior caval vein, *SCV* superior caval vein, *LI* left inferior, *LS* left superior, *RI* right inferior, and *RS* right superior pulmonary veins. (Reproduced from [14], with permission by AHA)

In reality, the site of the true septum is the area of the thin flap valve of the oval fossa and its apposition to the muscular rim (limbus) that surrounds it (Fig. 1b, c). During fetal life, the fossa valve allows blood to flow from the right atrium to the left atrium through the oval foramen (ostium secundum). After birth, the fossa valve completely adheres to the left atrial margin of the rim in most hearts, sealing the fossa opening. In about one quarter to one third of the normal population, there is probe patency of the oval fossa, even though the valve is large enough to overlap the rim. This is because the adhesion of the valve to the rim is incomplete, and this patency is the patent foramen ovale (PFO), which appears like a crescent-like edge on the left atrial aspect (Fig. 2a, d).

The superior rim of the fossa is then the infolded wall between the superior caval vein and the right pulmonary veins (Fig. 2b). Sandwiched between the fold are epicardial tissues, frequently containing the arterial supply to the sinus node. In some patients, the epicardial fat may increase the thickness of the infolding up to 1–2 cm. A thickness of >2 cm on noninvasive imaging is increasingly reported as indicative of lipomatous hypertrophy, with an incidence of up to 8% [15]. Even in hearts without so-called septal hypertrophy, transgression through the rim can hinder needle penetration and, being a thicker structure, can restrict maneuverability after crossing, besides increasing the risk of exiting the heart, dissecting into the fatty tissues, and causing hemopericardium [16]. At particular risk is the anterior rim of the fossa, which is adjacent to the aortic mound. The latter is seen as a protuberance of the atrial wall into the right atrial cavity. Because the aorta is just in front of the anterior walls of both atria, directing the puncture needle too anteriorly is likely to result in the needle entering the transverse pericardial sinus with a risk of aortic perforation (Fig. 2c, d). Owing to the more apical attachment of the tricuspid compared with the mitral valve at the septum level, the vestibule of the right atrium overlies the crest of the muscular ventricular septum (Fig. 2b). Consequently, the compact atrioventricular node situated on the slope of the crest is within 1 mm or so of the endocardial surface of the right atrium at the apex of the triangle of Koch [17].

The ideal site for crossing the septum to safely reach the left atrium is through the thin valve of the fossa. Although generally expected to be in the middle of the septal wall, the location and size of the oval fossa varies from patient to patient [18]. Furthermore, abnormalities of the thorax such as kyphoscoliosis or of the cardiovascular system like a marked left ventricular hypertrophy may result in displacement of the plane of the atrial septum and hence the oval fossa [19]. Patients with patches, PFO-occluder devices, thickly fibrosed septum that may be due to previous transseptal procedures, and aneurysmal valves [20] of the oval fossa are particularly challenging to perforate. The last of these is defined as a saccular excursion of >1 cm away from the plane of the atrial septum. In these hearts, the fossa membrane often is thinner, devoid of muscle cells, and mainly composed of connective tissue, making it more resistant to being perforated and yet more likely to be “tented” deep into the left atrial cavity with a greater risk of reaching the lateral wall.

Even with a transseptal puncture through the fossa valve, the relationship of the fossa to the superior wall or roof of the left atrium, the orifices of the pulmonary veins, and the mitral valve are important considerations for interventional procedures, especially in LAA closure procedure where the entrance of the needle should be directed toward the plane of the LAA ostium. In cases where the fossa is situated more superiorly than usual, the puncture site could lead to the atrial roof. The location is comparable to crossing at the site of a PFO, although the latter may also direct the catheter toward the anterosuperior wall of the left atrium (Fig. 2d), affecting ease of catheter handling and access to reach target areas. As will be described later, the LAA ostium could be more or less cephalad, anterior, or posterior; its assessment is essential to select the best position for transseptal puncture in order to align the device to the target area.

## The left atrium

**Fig. 3:**
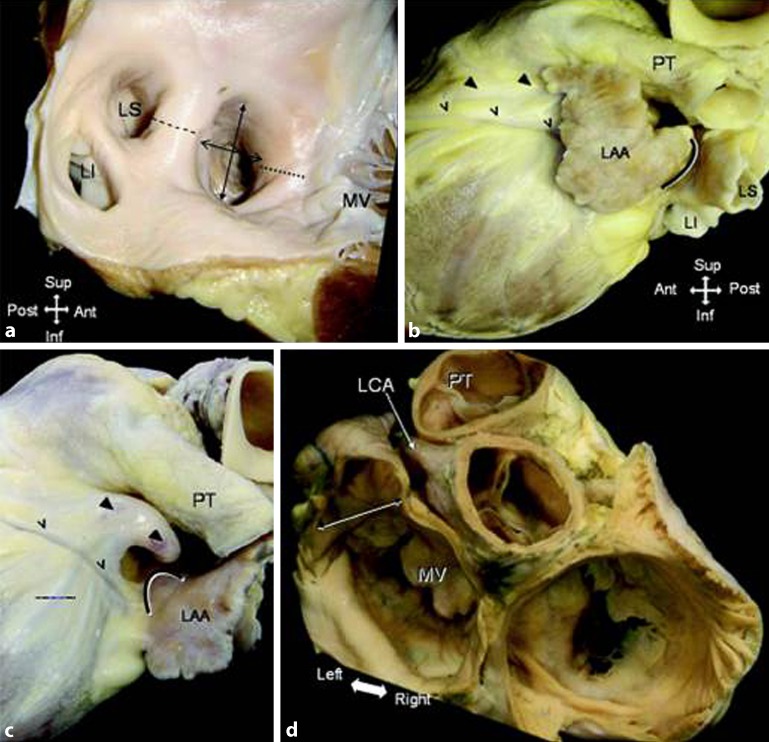
**a** Endocardial aspect of the left atrium showing the oval ostium of the appendage, the left lateral ridge separating it from the left inferior (*LI*) and superior (*LS*) pulmonary veins, and its proximity to the mitral valve (*MV*). **b,c** Left lateral view of the outside of a heart showing the narrow appendage overlying the course of the interventricular vein and artery (*v,* *arrowheads*) with its tip toward the right ventricular outflow tract. The appendage is deflected backward in **c**. **d** The ostium of the appendage (*double arrows*) in relation to the left coronary artery (*LCA*). *PT* pulmonary trunk, *LAA* left atrial appendage. (Reproduced from [32], with permission from BMJ Publishing Group Ltd.)

Following the direction of blood flow inside the left atrium, the atrial chamber begins at the pulmonary veno-atrial junctions and terminates at the fibro-fatty tissue plane that marks the atrioventricular junction at the mitral orifice. A distinctive appendage, considerably smaller than its counterpart on the right side, extends from the aforementioned main body of the atrium (Fig. 3). Apart from the appendage, which has a fairly well-defined opening (the os to the appendage), the other component parts of the atrium including the septal aspect do not have clear anatomic demarcations. The flap valve of the oval fossa occupies the septal aspect and it overlaps the fossa rim that is on the right atrial side. A crescentic mark at the anticipated site of the PFO is the anterocephalad margin of the valve membrane (Fig. 2a). If transseptal access is gained by passing through this tunnel-like opening from the right atrium, it is worth noting that the direction of the catheter on entering the left atrium is toward the anterocephalad wall, which often is particularly thin.

The posterior part of the left atrium receiving the pulmonary veins is its venous component. Mainly, the wall is smooth on the endocardial surface, without a clear distinction between venous and atrial walls, especially where the terminal parts of the veins are funnel-shaped. One of the most important aspects is that the posterior left atrial wall is not uniform in thickness. The transmural musculature of the wall shows changes in orientation of the myocardial strands; usually the areas of abrupt change are accompanied by a change in wall thickness [21].

Viewed from within the atrial cavity, the endocardial surface has the appearance of ridges in between the superior and inferior venous orifices. In addition, there is a ridge-like structure between the entrance of the left superior pulmonary vein and the os of the LAA, first described by Keith in 1907 [22] and today better known as the Coumadin ridge [23] or left lateral ridge. This “ridge” is a fold that has thicker muscle in the anterosuperior portion and within it runs the remnant of the vein of Marshall, abundant autonomic nerve bundles, and a small atrial artery that, in some cases, is the sinus nodal artery [24].

Passing across the subepicardium of the anterior left atrial wall is Bachmann’s bundle, also known as the interauricular band, which is the most prominent muscular interatrial bridge [25]. Myocardial strands from its superior rightward arm can be traced toward the location of the sinus node and the terminal crest. Leftward, the bundle runs toward the LAA where it branches, passing to either side of the appendage and then reuniting to continue into the musculature of the lateral and postero-inferior atrial walls [26].

## The left atrial appendage

In most hearts the appendage extends from between the anterior and lateral walls of the left atrium and its tip is directed anterosuperiorly, overlapping the left border of the right ventricular outflow tract or the pulmonary trunk and the main stem of the left coronary or the circumflex artery (Fig. 3). It is not uncommon to find the tip of the appendage directed laterally and backward, while in a few hearts the tip portion passes behind the arterial pedicle to sit in the transverse pericardial sinus. There is broad variability in LAA morphology, a fact that complicates adequate evaluation. Several studies have described the LAA as a long tubular and hooked structure with different lobes. In one study based on computed tomography (CT) and magnetic resonance imaging (MRI), the LAA was classified into four morphological groups: the cactus, with a dominant central lobe with secondary lobes extending from the central lobe in both superior and inferior directions; the chicken wing, with a bend in the proximal or middle part of the dominant lobe and sometimes secondary lobes; the windsock, with one dominant lobe of sufficient length as the primary structure; the cauliflower, with limited overall length and more complex internal characteristics with a variable number of lobes without a dominant one. The more lobes and pouches the LAA has, the higher the risk of thrombus formations inside for AF patients [27]. However, this classification of the morphology should be made with caution because imaging the same appendage from different perspectives can change its appearance [28].

**Fig. 4:**
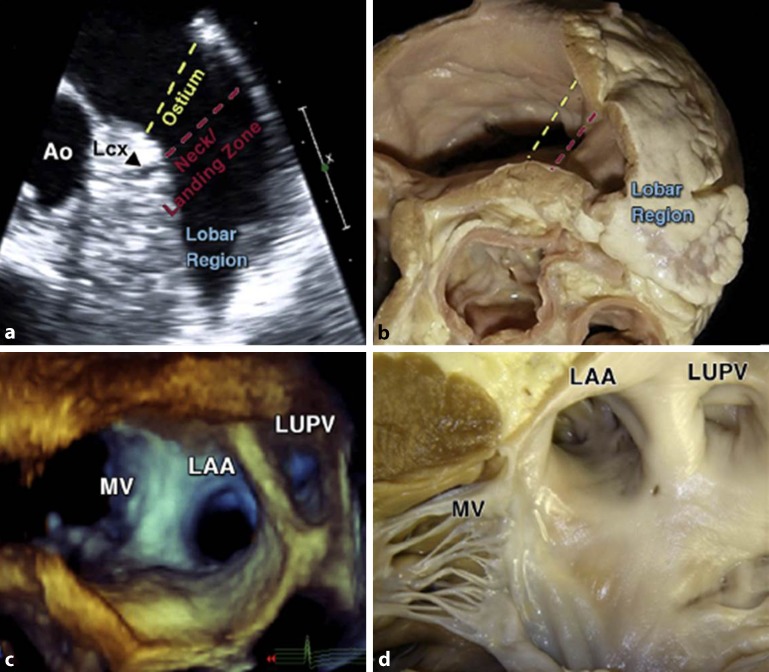
**a** Left atrial appendage (*LAA*) regions are illustrated in a two-dimensional transesophageal echocardiography (TEE) view (45°). The *black arrowhead* indicates the circumflex artery (*Lcx*). **b** Corresponding anatomic image. **c** Three-dimensional TEE reconstruction of the relationship between the LAA, the left upper pulmonary vein (*LUPV*), and the mitral valve (*MV*). **d** Corresponding anatomic view. *Ao* aorta. (Reproduced from [5], with permission by Elsevier)

As shown in Fig. 4, it is also useful to describe the LAA according to three regions: the ostium, the neck (or landing zone for devices), and the lobar regions. Within the appendage are pectinate muscles. Unlike the pectinate muscles in the right atrium, however, the pectinate muscles in the left atrium do not arise from a distinct muscle bundle that is like a crista terminalis. Instead, they present with coconut-palm leaf arrangement especially at the borders between superior and inferior surfaces, or are strap-like, or resemble a palmyra-palm leaf arrangement near the border with the atrial vestibule [29]. On imaging, the thicker bundles may be mistaken for thrombi or intra-atrial masses [30]. The remainder of the LAA wall in between the muscle bundles is exceptionally thin.

Patients with chronic AF frequently have LAA remodeling in which there is dilation, stretching, and reduction in pectinate muscle volume, as well as endocardial fibroelastosis [31]. Because some LAA morphologies are more challenging for device closure than others, careful evaluation of its anatomy is required before any planned procedure. A chicken-wing LAA morphology, characterized by an early (<20 mm from the ostium) and severe bend, is one of the most difficult anatomic variations for LAA closure [5]. A secondary lobe originating close to the ostium can pose problems, because it may not be covered after device deployment. Two large lobes of a similar size separated by a large rim in between them may cause problems if the remaining proximal portion of the LAA is too short to accommodate a device. A cone-shaped LAA with a progressive reduction in dimensions from its orifice to its distal tip might pose a particular problem for secure seating of the occlusion device that is further aggravated by the lack of trabeculations in the LAA landing zone region and hence increasing the risk of device migration or embolization. Furthermore, if a plug type of device is used, the chosen disc may be too small to adequately seal the ostium since it is considerably wider than the landing zone [5].

Previous studies [29, 32] showed that the shape of the ostium is mostly elliptical or oval (Fig. 3a). Round, teardrop, or triangular shapes are far less common. This suggests that to seal the LAA orifice adequately without oversizing, devices may need to be elliptical for a snug fit. The percutaneous devices systems, however, have a round shape to fill or cover the ostium. A round implant over an oval orifice may leave crevices on either side of the implant, leading to incomplete sealing of the orifice. Nevertheless, the significance of residual leaks after device implantation is not clear.

Ramondo et al. stated that the diameter of the ostium is in relation to the length of the LAA, which is a crucial measurement in order to permit complete deployment of the device into the LAA [33]. Another critical issue for the entry of the deployment catheter is the distance between the LAA orifice and the point at which the LAA first deviates from its original course. It has been reported that for deviations of between 7 and 12 mm from the ostium [32], if the deployment catheter is advanced too far it may easily exit the appendage into the pericardial space, especially since parts of its wall in between the pectinate muscles are paper-thin. The risk of a hemopericardium, as already mentioned, should not be overlooked [34].

Aside from the paper-like thickness of the LAA wall itself, the morphology of the atrial wall in the proximity of the LAA orifice is also an important consideration when deploying catheters in the left atrium. In almost 50% of heart specimens there are pits or troughs that tend to occur in isolation or in clusters located on the anterolateral and lateral atrial wall [32]. Interventionists should be aware that, as during maneuvers, catheters and delivery sheaths may become lodged in the pits/troughs. This could potentially increase the risk of perforation since the atrial wall is extremely thin inside the depressions, comparable to the paper-thin areas within the LAA.

**Fig. 5:**
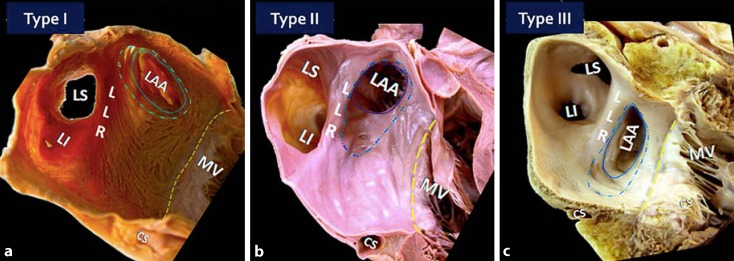
Classification of the left atrial appendage (*LAA*): *Type* *I*, with the LAA ostium cephalad and anterior to the left superior pulmonary vein (*LS*) and usually separated by a wide lateral ridge (*LLR*); *Type* *II*, when the most posterior part of the ostium is very close to the lateral ridge, which is thinner and more marked than in type I and demarcates a well-defined limbus; *Type* *III* in which the ostium is at the level of the left inferior pulmonary vein (*LI*). *CS* coronary sinus, *MV* mitral valve. (Reproduced from [35], with permission by Wiley)

Finally, we should not forget that the entrances of the pulmonary veins, the atrial appendage, and part of the left atrial body are in close vicinity to important structures surrounding the heart (Figs. 3 and 4). The introduction of LAA closure devices has increased the need for knowledge of subtler anatomical aspects that might be very useful for understanding possible difficulties during the device implantation, such as its relation with the left pulmonary veins and the length and depth of the lateral ridge as well as the distance to the mitral valve. These anatomical structures are important for a better spatial understanding of the LAA ostium and neck and could guide the approach for device delivery in each individual [35]. A classification suggested by López-Mínguez and colleagues [35] of the “LAA ostium” (which corresponds to the neck region in other publications) according to its relationship with the pulmonary veins is as follows:

- Type I, with the LAA ostium (neck) cephalad and anterior to the left superior pulmonary vein and usually separated by a wide lateral ridge (LLR)
- Type II, when the most posterior part of the ostium is very close to the lateral ridge, which is thinner and more marked than in type I and demarcates a well-defined limbus
- Type III in which the ostium is at the level of the left inferior pulmonary vein (Fig. 5).

CMR or CT studies before intervention may help to classify the different types of ostium (or neck) and therefore may be useful for selecting the devices and for the site in which to perform the transseptal puncture. Type I is probably the most suitable for device occlusion since the broad LLR could favor a stable implant as well as having more distance from the pulmonary vein. By contrast, types II and III could present with an extremely narrow and pointed LLR, in which case it could be more challenging to achieve a balance between device stability without risking pulmonary venous obstruction if a larger device is deployed [35].

Apart from the left pulmonary veins in proximity to the ostium of the appendage, the mitral valve is also in its vicinity in hearts where there is a narrow vestibule. An oversized device may impinge on the mitral orifice.

Moreover, it is also important to consider neighboring outer structures whether attempting to close the LAA ostium from within the atrium or from the pericardial space. Owing to its slightly flattened shape, the lower surface of the LAA usually overlies the summit of the left ventricle while the upper surface is beneath the fibrous pericardium. A previous study [32] showed that the left anterior descending coronary artery and the circumflex artery are in close proximity to the LAA or its ostium and can be vulnerable to trauma during implantation of percutaneous devices, especially when devices are 20–40% larger than the os. It is well established that the sinus node artery arises from the right coronary system in about 60% of cases. However, there is still a significant proportion of cases in which the sinus node artery originates from the left coronary system. In the landmark study by Busquet et al., the sinus node artery was seen to arise from the circumflex artery and from the left lateral atrial artery in 30% and 8% of the cases, respectively [36]. When these run around the ostium of the LAA, they can be at risk of trauma from devices.

A previous anatomical study on the course of the phrenic nerves has demonstrated that the left phrenic nerve runs along the pericardium overlying the LAA [37]. This nerve may also be at risk if epicardial approaches are used. Moreover, on the epicardial aspect, the anterior interventricular trunk and the obtuse marginal trunk join to form the left coronary lymphatic channel that passes beneath the LAA and close to the ostium [38]. The great cardiac vein on its ascent to the atrioventricular groove also passes underneath the appendage but its course tends to veer away from the ostium. Therefore, the observations concerning the spatial relationships of structures and the atrial wall are still highly relevant and important when contemplating occlusion or exclusion of the LAA.

## Practical conclusion

Percutaneous LAA closure is a relatively new, but evolving treatment strategy to prevent embolic events in patients suffering with nonvalvular AF. Sound knowledge of atrial anatomy from both endocardial and epicardial aspects is necessary. This knowledge will help interventionists take advantage of imaging techniques when assessing the suitability of the LAA anatomy for the closure, for selecting the optimal device types and sizes, and for guiding the LAA closure procedure in order to reduce complications and increase procedural success.
